# The effect of the Mid-Day Meal programme on the longitudinal physical growth from childhood to adolescence in India

**DOI:** 10.1371/journal.pgph.0002742

**Published:** 2024-01-11

**Authors:** Shivani Gharge, Dimitris Vlachopoulos, Annie M Skinner, Craig A Williams, Raquel Revuelta Iniesta, Sayeed Unisa

**Affiliations:** 1 Department of Mathematical Demography and Statistics, International Institute for Population Sciences, Mumbai, Maharashtra, India; 2 Children’s Health and Exercise Research Centre, Faculty of Health and Life Sciences, University of Exeter Medical School, University of Exeter, Exeter, United Kingdom; PLOS: Public Library of Science, UNITED STATES

## Abstract

The study aims to examine the effect of the world’s largest school-feeding programme, the Mid-Day Meal (MDM) programme, on the changes in the underweight prevalence among school-children in India. Data from the Indian Human Development Survey (IHDS) Rounds 1 (2004–05) and 2 (2011–12) were utilized. The sample included individual-level information of children aged 6 to 9 years in IHDS-1 who then turned 13 to 16 years in IHDS-2. The sample was categorised into four groups based on their MDM consumption history (Group 1: no MDM support in IHDS-1 and IHDS-2, Group 2: MDM support in IHDS-1, Group 3: MDM support in IHDS-2, Group 4: persistent MDM support in IHDS-1 and IHDS-2). The dependent variable was underweight status as defined by the World Health Organisation Child Growth Standards Body Mass Index for age (BMI Z-score) < -2 SD of the median. Bivariate analysis was used to examine the prevalence of underweight and establish associations between underweight status and socio-demographic characteristics. Logistic regression was performed to assess the strength of the association of socio-demographic characteristics and MDM consumption patterns with underweight across poor and non-poor asset groups. The findings suggest that early and persistent MDM support among respondents reduced the likelihood of low BMI Z-scores compared to those without MDM support. Respondents from the poor asset group who received MDM support in at least one of the two survey rounds had higher odds of being underweight in comparison with those who did not receive MDM support at all. Girls and adolescents residing in the Eastern region of India were less likely to be underweight. The study shows that the MDM programme was effective in reducing the rate of underweight among school children. However, continuous programme upscaling with a special focus on children from poor households will significantly benefit India’s school-aged children.

## Introduction

Childhood to adolescence is a period of rapid growth and development, which is highly dependent on optimal nutrient and energy intake [1]. Inadequate dietary intake during this critical period of growth may lead to undernutrition. Undernutrition can cause irreversible stunting, higher risk of infections and compromise organ development including the brain, which may affect physical, emotional and social wellbeing [2]. Therefore, nutritional interventions targeting school-aged children have a significant impact on health and assist in uplifting physical and/or mental health benefits, thereby improving children’s chances for a better future [3].

Undernutrition across various socioeconomic strata is the most challenging issue faced by children and adolescents all over the world [4]. Body Mass Index (BMI) is a measure of acute nutritional status and is currently considered the gold standard to assess nutritional status in children and adolescents [5–7]. According to World Health Organization (WHO) child growth standards, children, and adolescents whose BMI Z-score is below -2 SD are considered underweight [5, 6]. The prevalence of children and adolescents who were underweight declined from 9.2% in 1975 to 8.4% in 2016 among girls and from 14.8% to 12.4% among males [8]. Globally, around 75 million girls and 117 million boys were moderately or severely underweight in 2016 [9]. The prevalence of underweight among children and adolescents was highest in India in 2016, at 22.7% among girls and 30.7% among boys, and it has not decreased substantially in the last three decades [8]. According to the Comprehensive National Nutrition Survey (CNNS) India 2016–18 report, 10% of school-age (5–9 years) children and 47% of late adolescent girls aged 15–19 years were underweight in India [10]. Undernutrition significantly compromises optimal growth and development of children and is associated with a higher risk of infectious diseases, reducing the ability to learn, lowering school performance [11, 12]. In the long term, it increases the risk of non-communicable diseases e.g., cardiovascular diseases, diabetes and osteoporosis, is associated with adverse pregnancy outcomes and importantly affects economic productivity [12, 13].

In 1995, the Government of India launched the National Programme of Nutritional Support to Primary Education (NSPE) as a centrally sponsored scheme with the primary objective of improving enrolment, retention, and attendance with a simultaneous effort to improve the nutritional status of schoolchildren [14]. On November 28, 2001, the Supreme Court directed all the State Governments/Union territories to implement the Mid-Day Meal (MDM) Scheme, in which every child in every Government and Government aided school was to be served a cooked meal with a minimum content of 300 kilocalories and 8–12 gram protein per day for a minimum of 200 days per year [14]. The majority of the Indian states began providing cooked and warm meals by 2003, and eventually, around 120 million students were covered under the MDM by 2006, which is now regarded as the world’s largest school feeding programme [15].

The fundamental aim of this programme is to increase school enrolment, retention, and attendance of children in India by providing free cooked meals for lunch on working days to children in primary and upper primary classes in government, government-aided, local body, Education Guarantee Scheme. Alternate innovative education centres, Madrassa and Maqtabs supported under Sarva Shiksha Abhiyan, and National Child Labour Project Scheme schools run by the Ministry of Labour and Employment are also included [14]. Since the inception of MDM, the programme has undergone several transformations before reaching the current phase. The MDMs appear to have substantially contributed to overcoming classroom hunger, and have been a huge help to low-income families, relieving them of the responsibility of providing a one-time meal to their children [16, 17]. Universal primary education has been achieved in the last decade, but enrolment has only improved marginally [18]. The MDM programme has led to an increase in school participation rate from marginalised households [18], an improvement in dietary and total energy intake (TEI) during school days only [16, 17] and improvement in weight-for-age (WFA) and height-for-age (HFA) [19, 20].

Benefits from the MDM programme have shown significant “improvement” in WFA and HFA in children during severe droughts [20]. However, MDM’s longitudinal effects on children’s physical growth as pupils transition from MDM beneficiaries to non-beneficiaries and vice versa, has not been investigated [21], especially at the national level. Therefore, this study sought to add new knowledge to the existing research and contribute to the larger literature on school feeding, with a special focus on the effect of school feeding on underweight. Employing a longitudinal data set from Rounds 1 and 2 of the Indian Human Development Survey (IHDS-1 and 2), the present study aimed to examine the changes in the prevalence of underweight (defined as BMI-for-age < -2 SD of the WHO Child Growth Standards median) in schoolchildren aged 6 to 9 years in IHDS-1 and 13 to 16 years in IHDS-2 as they transition from MDM beneficiaries to non-beneficiaries and vice versa. The secondary aim was to examine the prevalence of underweight status according to different socio-demographic characteristics and determine the predictors of underweight.

## Ethics statement

This study employed a publicly available longitudinal secondary dataset that had no information that may lead to the respondents’ identification. The Inter-University Consortium for Political and Social Research (ICPSR) data repository retains all the IHDS datasets that was utilised in this study [22, 23].

## Materials and methods

### Data source

India has one of the largest education systems in the world, with more than 1.4 million schools, 9.7 million teachers, and 265 million children [24]. To understand the effect of the MDM programme on underweight in India, we utilise data from the Indian Human Development Survey (IHDS). The IHDS is an initiative through a collaborative programme by researchers from the National Council of Applied Economic Research, New Delhi, and the University of Maryland. The data for Round 1, IHDS-1, was collected in 2004–05, and for Round 2, IHDS-2, it was collected in 2011–12. The survey yields information pertaining to the key dimensions of human development indicators and a series of quantifiable variables measuring wider contexts. A nationally representative, multitopic survey encompassing 41,554 households from 1,504 villages and 970 urban neighbourhoods in Round 1 [22] and 42,152 households from 1503 villages and 971 urban neighbourhoods in Round 2 [23] across India. Around 83%, i.e., 34,621 households, were re-interviewed in Round 2 along with some split households residing in the same community. IHDS collects extensive information on sociodemographic characteristics, education, fertility, health, agriculture, energy use, and utilisation of large public programmes like the Integrated Child Development Services and Public Distribution System at the national level.

### Study design

**Fig 1:**
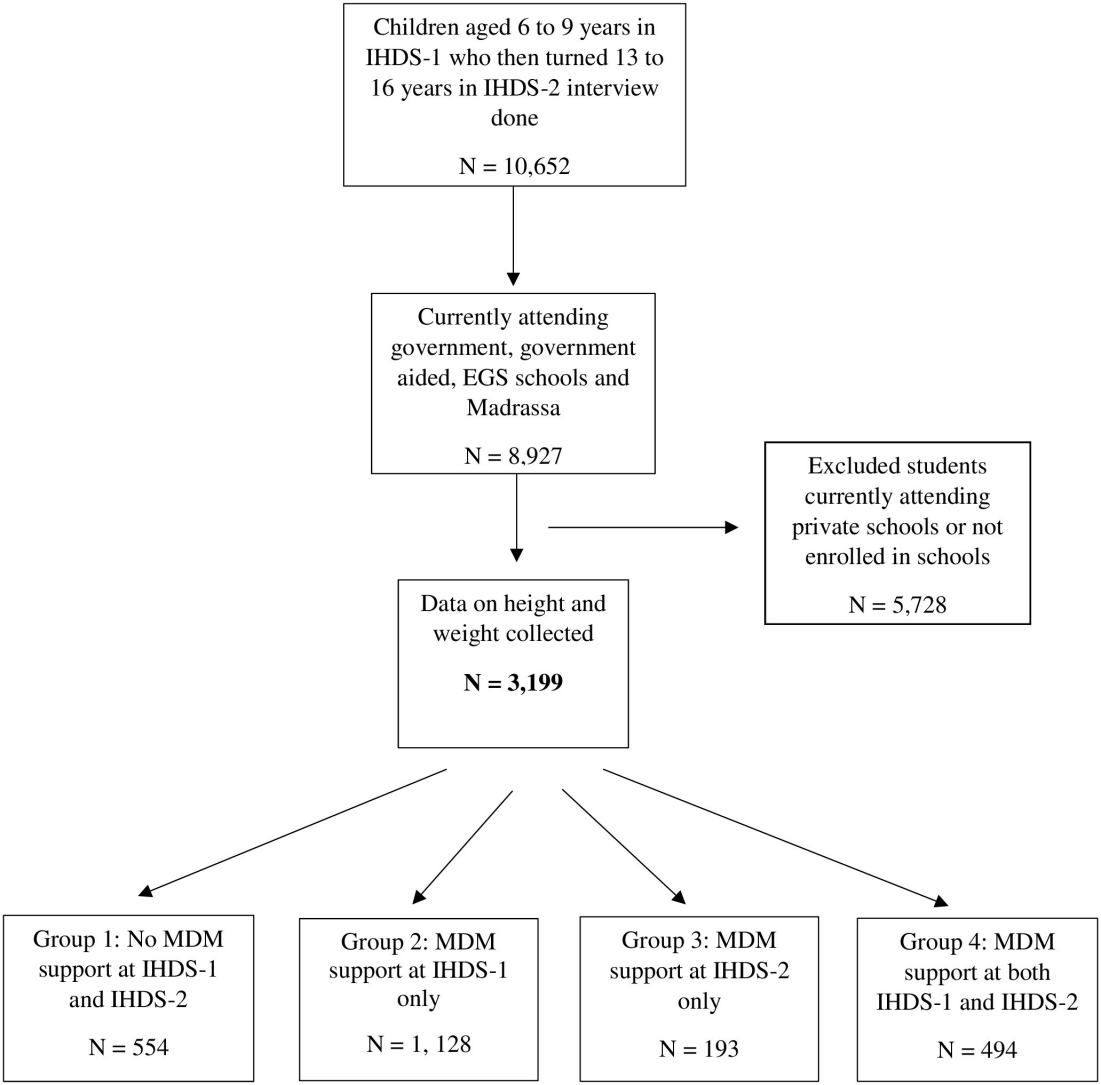
Eligibility criteria for the study sample, IHDS-1 (2004–2005) and IHDS-2 (2011–2012). (A) Details of the final sample of re-interviewed respondents and the sample selection process are presented in this figure; (B) Abbreviations: IHDS, Indian Human Development Survey; N: Frequency; EGS, Education Guarantee Scheme; MDM, Mid-Day Meal.

We utilized panel data from Rounds 1 (2004–05) and 2 (2011–12) of the IHDS, and our final sample size was 3,199 (1,638 girls and 1,561 boys). For the analytical purposes of our study, data was restricted to the individual-level information of children aged 6 to 9 years in IHDS-1 who then turned 13 to 16 years in IHDS-2 and were currently attending government, government-aided, EGS, and Madrassa schools. We included schoolchildren from the above-mentioned age-group because the MDM scheme was covering primary school children (classes I to IV) and since 2008 the programme covers all children studying in Government, Local Body and Government-aided primary and upper primary schools and the Education Guarantee Scheme schools or alternate innovative education centres including Madrassa and Maqtabs supported under SSA of all areas across the country [14]. The percentage of loss to follow-up for all the observations was 17% [22, 23], and for our study sample it was 25%, which was very low; therefore, we excluded individuals lost to recontact for IHDS-2 from the study sample. Details of the final sample of re-interviewed respondents and the sample selection process are presented in Fig 1 (also see [22, 23, 25, 26] for further details on the sample of re-interviewed households).

### Variable description

#### Outcome variable

The main outcome was underweight defined as BMI-for-age (BMI Z-score). BMI Z-score is currently considered the gold standard to assess nutritional status in children and adolescents [5, 6]. Nutritional status is defined as underweight (BMI Z-score < -2 SD), normal weight (BMI Z-score -2 SD to ≤ 1 SD), overweight (> 1 SD to ≤ 2SD) and obese (BMI Z-score > 2SD). Data on the height and weight of the respondents were recorded in IHDS-1, and followed up in IHDS-2. BMI-for-age Z-scores were calculated, and the results were classified as underweight if their BMI-for-age was more than two standard deviations below (< -2 SD) the WHO Child Growth Standards median [5, 6].

#### Explanatory variables

In IHDS-1, MDM consumption was defined as those who received grain, Dalia and/or a variety of meals. In IHDS-2, MDM consumption was defined as those who received school meals regularly and irregularly. The participants were categorized into four groups according to their response to the question on MDM consumption from IHDS-1 and IHDS-2; group one (No MDM support in IHDS-1 and IHDS-2), group two (MDM support in IHDS-1 only), group three (MDM support in IHDS-2 only), and group four, (MDM support in both IHDS-1 and IHDS-2).

The socio-demographic characteristics include sex, household size stratified as ≤ 4 members, 5 to 8 members and ≥ 9 members, asset groups (poorest, poor, middle and rich) [27, 28], household adult’s education (all are illiterate, at least one completed primary, at least one completed secondary, at least one completed higher), place of residence (urban and rural), religion (Hindu, Muslim, Christian and other) and region (North, Central, East, Northeast, West, South).

### Statistical analyses

Descriptive statistics were calculated to show the mean height (cm), weight (kg) and BMI Z scores of the study sample. Bivariate analyses were performed to estimate the prevalence of underweight (BMI Z score < -2 SD) among children and adolescents aged 6 to 9 years in IHDS-1 who then turned 13 to 16 years in IHDS-2 in India by transition in MDM consumption as well as for the categories of independent variables. Chi-square tests were used to determine whether independent variables, sex, household size, asset group, household adult’s education, place of residence, religion and region had significant associations with the prevalence of underweight status at p < 0.05. The asset group has not changed over time from IHDS-1 (2004–2005) to IHDS-2 (2011–2012) which is why we have considered only the sample from IHDS-2 (2011–2012) in our multivariate analysis (Table 3). Two separate multivariate models were then fitted to discern the extent to which poor and non-poor asset groups, and in combination with MDM consumption pattern and sociodemographic factors, explain the associations with underweight (BMI Z score < -2 SD). Model 1 is controlled for MDM consumption pattern, socio-demographic variables and poor asset group category, whereas, Model 2 is controlled for MDM consumption pattern, socio-demographic variables and non-poor asset group category.

The equation for the logistic regression is as follows:

$$\text{ln}\frac{P_{i}}{\left(1-\;P_{i}\right)}=\beta_{0}+\;\beta_{1}\;x_{1}+\;\beta_{2}\;x_{2}+\cdots \;\beta_{K}\;x_{k}$$ (1)

Where β_0_ … β_K_ are regression coefficients indicating the relative effect of a particular explanatory variable on the outcome variable. Analysis of variance (ANOVA) was used to determine significant change in BMI Z scores from IHDS-1 to IHDS-2 within the four groups and whether that change was different between the groups. Further, individual weights were used to make the estimates nationally representative. The data analyses were performed using STATA version 15.0 (StataCorp; College Station, TX, USA). The nature of the primary outcome variable required the above-mentioned approach of statistical analysis following the suggestion of a statistician to allow better interpretation of the data.

## Results

### Socio-demographic characteristics of the study population

Descriptive characteristics of the study sample are shown in Table 1. In this study sample of 3,199 respondents, 50% of the study participants were girls (IHDS-1: n = 1638, 50.45%; IHDS-2: n = 1638, 50.31%) and 50% were boys (IHDS-1: n = 1561, 49.55%; IHDS-2: n = 1561, 49.69%). Around 64% (n = 2088) and 61% (n = 2030) of the study participants had a household size of 5 to 8 members in IHDS-1 and IHDS-2 respectively. At least one adult member in 46% to 48% of the households had completed secondary level education. The highest proportion of study participants were Hindu by religion (IHDS-1: n = 2719, 85.87%; IHDS-2: n = 2710, 85.25%). Majority of the sample resided in rural areas with 81% (n = 2453) rural sample in IHDS-1 and 85% (n = 2452) rural sample in IHDS-2. The Northern region formed the bulk of the study sample, followed by the Eastern and Western regions.

**Table 1:** Socio-demographic characteristics and growth outcomes of the study population, IHDS-1 (2004–2005) and IHDS-2 (2011–2012).

Background Characteristics	Total (N = 3,199)	
	% (N)	
	IHDS-1 (2004–2005)	IHDS-2 (2011–2012)
**Sex**		
Boys	49.55 (1561)	49.69 (1561)
Girls	50.45 (1638)	50.31 (1638)
**Household size**		
Less than or equal to 4 members	14.50 (469)	27.22 (820)
5 to 8 members	63.69 (2088)	60.92 (2030)
Greater than or equal to 9 members	21.82 (642)	11.86 (349)
**Asset group**		
Poorest	24.62 (670)	25.22 (653)
Poor	23.85 (707)	22.71 (693)
Middle	25.21 (794)	26.18 (896)
Rich	26.33 (1028)	25.89 (956)
**Education of adult members in the HH** *(N = 3*,*194 in IHDS-1)*		
All are illiterate	25.94 (790)	24.09 (719)
At least one completed Primary	11.06 (308)	7.67 (243)
At least one completed Secondary	46.40 (1575)	48.52 (1597)
At least one completed Higher	16.60 (521)	19.72 (640)
**Religion**		
Hindu	85.87 (2719)	85.25 (2710)
Muslim	12.82 (423)	13.51 (433)
Christian and other	1.31 (57)	1.24 (56)
**Place of residence**		
Rural	81.01 (2453)	77.50 (2452)
Urban	18.99 (746)	22.50 (747)
**Region**		
North	33.39 (1173)	33.97 (1173)
Central	9.92 (413)	9.45 (413)
East	25.35 (593)	24.58 (593)
Northeast	1.96 (78)	2.03 (78)
West	16.60 (502)	15.72 (502)
South	12.78 (440)	14.24 (440)
	**Mean (SD)**	
Height (cm)	116.83 (12.72)	151.79 (10.75)
Weight (kg)	20.54 (8.04)	42.04 (8.15)
BMI (kg·m^2^) Z scores	-0.90 (1.61)	-0.91 (1.31)

Note: (a) For some variables the ‘N’ is not additive to the total ‘N’ because of missing cases.

(b) N: Frequency.

(c) IHDS, Indian Human Development Survey.

(d) %: Percentage;

(e) SD: Standard deviation.

(f) HH: Household.

(g) BMI: Body Mass Index.

Table 2 shows the underweight prevalence among school-aged children by socioeconomic characteristics and the change in MDM consumption status from Rounds 1 and 2 of the IHDS. The findings indicate that underweight prevalence is highest among school-aged children who have received MDM support at an early age of 6 to 11 years in IHDS-1 (IHDS-1: 22.36%; IHDS-2: 18.40%), and among those who have received persistent MDM support (IHDS-1: 22.31%; IHDS-2: 21.68%). The highest decline of around 4% in underweight prevalence can be observed among those respondents who received MDM support during their early ages. In group 1 (No MDM support at IHDS-1 and IHDS-2) there is a statistically significant relationship between underweight status and sex, household size and region. In group 2 (MDM support at IHDS-1 only), underweight status is dependent on sex, education of adult members in the household and region. In group 4 (MDM support at IHDS-1 and IHDS-2), underweight status is significantly associated with region.

**Table 2:** Underweight prevalence among school-aged children by socioeconomic characteristics and change in MDM consumption status from IHDS-1 (2004–2005) to IHDS-2 (2011–2012).

Background characteristics	Underweight prevalence by change in MDM consumption status (%)															
	Group 1: No MDM support at IHDS-1 and IHDS-2				Group 2: MDM support at IHDS-1 only				Group 3: MDM support at IHDS-2 only				Group 4: MDM support at IHDS-1 and IHDS-2			
	IHDS-1	p-value	IHDS-2	p-value	IHDS-1	p-value	IHDS-2	p-value	IHDS-1	p-value	IHDS-2	p-value	IHDS-1	p-value	IHDS-2	p-value
Total*	15.75		14.98		22.36		18.4		20.69		19.62		22.31		21.68	
**Sex**																
Boys	16.51	0.441	20.86	< 0.01	24.68	0.368	24.79	< 0.001	24.63	0.742	26.12	0.11	22.48	0.458	23.94	0.028
Girls	14.89		7.93		19.94		11.78		16.63		13.42		22.16		19.54	
**Household size**																
Less than or equal to 4 members	16.53	0.298	6.05	< 0.05	19.65	0.993	15.64	0.271	19.36	0.904	14.52	0.792	28.21	0.305	30	0.243
5 to 8 members	16.91		20.16		23.51		18.28		21.95		22.62		21.54		19.21	
Greater than or equal to 9 members	11.66		15.16		20.91		25.26		17.59		14.22		20.79		15.47	
**Asset groups**																
Poorest	7.72	0.668	9.36	0.577	28.33	0.076	19.6	0.145	27.91	0.078	26.39	0.341	26.03	0.091	22.77	0.286
Poor	18.47		5.34		20.92		20.66		22.73		15.38		20.11		17.06	
Middle	14.44		25.83		24.52		16.73		17.88		22.42		20.97		26.72	
Rich	17.61		13.91		17.61		17.28		14.36		12.07		19.86		19.44	
**Education of adult members in the HH**																
All are illiterate	4.46	0.348	10.8	0.959	25.85	0.819	20.69	< 0.05	22.27	0.747	10.83	0.424	22.08	0.185	17.26	0.536
At least one completed Primary	15.23		7.93		23.81		8.61		18.66		29.15		32.7		33.18	
At least one completed Secondary	21.35		20.04		21.84		18.85		22.99		17.94		19.8		23.46	
At least one completed Higher	12.9		10.24		18.89		17.89		11.56		28.82		21.33		17.4	
**Religion**																
Hindu	15.28	0.102	15.66	0.57	22.58	0.44	18.5	0.778	22.98	0.181	20.48	0.38	22.38	0.474	21.27	0.308
Muslim	21.64		11		22.76		18.02		3.78		15.7		22.72		24.88	
Christian and others	7.53		9.09		2.38		9.3		-		-		-		-	
**Place of residence**																
Rural	15.36	0.408	17.53	0.197	22.76	0.361	19.21	0.102	21.97	0.833	19.74	0.679	22.49	0.699	20.66	0.905
Urban	16.57		10.24		20.15		14.9		16.57		19.31		20.73		29.48	
**Region**																
North	13.28	< 0.05	20.92	0.207	18.94	< 0.01	20.73	< 0.001	12.86	0.13	27.52	0.061	21.63	< 0.001	18.35	< 0.05
Central	22.47		20.6		17.22		21		10.93		52.19		8.82		25.99	
East	6.84		6.26		23.89		9.68		21.25		14.46		21.56		15.52	
Northeast	36.65		5.17		12.12		7.13		60.91		5.06		74.24		-	
West	17.45		12.42		32.83		22.78		32.46		9.36		40.71		34.62	
South	36.43		9.98		16.12		18.65		19.52		19.88		17.84		20.91	

Note: (a) Respondent was considered underweight if BMI-for-age was more than two standard deviations below (< -2SD) the WHO Child Growth Standards median.

(b) P-values are for the Chi-square tests and p-values < 0.05, < 0.01 and < 0.001 indicate significant association between the dependent and independent variables.

(c) %: Percentage.

(d) Abbreviations: MDM, Mid-Day Meal; IHDS, Indian Human Development Survey; HH: Household.

Tables 3 and 4 show whether there is a significant increase or decrease in BMI Z scores from IHDS-1 to IHDS-2 within the four groups and whether that change is different between the groups. The ANOVA tests show that there was a significant decrease in BMI Z scores from IHDS-1 to IHDS-2 within group 3 (-0.46 ± 1.87 for IHDS-1 and -0.93 ± 1.39 for IHDS-2) and 4 (-0.86 ± 1.61 for IHDS-1 and -1.1 ± 1.37 for IHDS-2). In case of boys, there was a significant decrease in BMI Z scores from IHDS-1 to IHDS-2 within groups 2 (-1.00 ± 1.53 for IHDS-1 and -1.19 ± 1.26 for IHDS-2), 3 (-0.19 ± 2.08 for IHDS-1 and -1.02 ± 1.47 for IHDS-2) and 4 (-0.81 ± 1.73 for IHDS-1 and -1.21 ± 1.47 for IHDS-2). The ANOVA also confirms that the change in the BMI Z scores is also different between the groups. Respondents from group 2 (MDM support at IHDS-1 only) and 4 (MDM support at IHDS-1 and IHDS-2) are 36% and 39% significantly less likely to have a low BMI Z score as compared to respondents from group 1 (No MDM support at IHDS-1 and IHDS-2).

**Table 3:** Analysis of variance for BMI Z scores in IHDS-2 and MDM consumption status from IHDS-1 to IHDS-2.

Change in MDM consumption status	BMI Z scores in IHDS-2	
	Coef.	[95% CI]
**Group 1: No MDM support**		
**Group 2: Early age MDM support**	-0.36***	[-0.47 - -0.26]
**Group 3: Late age MDM support**	-0.11	[-0.29 - 0.06]
**Group 4: Persistent MDM support**	-0.39***	[-0.52 - -0.26]

Note: (a) Coef.–Coefficient.

(b) 95% Confidence Interval is given in parenthesis.

(c) ANOVA (Null hypothesis: Mean BMI Z score values change from IHDS-1 to IHDS-2 were not different among the 4 groups).

(d) Statistical significance denoted by asterisks: *, **, *** for p-value<0.05, p-value<0.01, p-value<0.001 respectively, reject null hypothesis-there is a significant difference between the groups.

(e) Abbreviations: MDM, Mid-Day Meal; BMI, Body Mass Index; IHDS, Indian Human Development Survey

**Table 4:** Analysis of variance for BMI Z scores by sex and change in MDM consumption status from IHDS-1 to IHDS-2.

Sex differential	BMI Z score Mean (SD)											
	Group 1: No MDM support			Group 2: MDM support at IHDS-1 only			Group 3: MDM support at IHDS-2 only			Group 4: MDM support at IHDS-1 and IHDS-2		
	IHDS-1	IHDS-2	ANOVA p-value	IHDS-1	IHDS-2	ANOVA p-value	IHDS-1	IHDS-2	ANOVA p-value	IHDS-1	IHDS-2	ANOVA p-value
**Total**	-0.54 (1.72)	-0.64 (1.26)	0.2776	-0.93 (1.55)	-0.97 (1.23)	0.6140	-0.46 (1.87)	-0.93 (1.39)	0.0012	-0.86 (1.61)	-1.1 (1.37)	0.0068
**Boys**	-0.53 (1.80)	-0.73 (1.33)	0.1044	-1.00 (1.53)	-1.19 (1.26)	0.0267	-0.19 (2.08)	-1.02 (1.47)	p < 0.001	-0.81 (1.73)	-1.21 (1.47)	0.0043
**Girls**	-0.55 (1.63)	-0.55 (1.18)	0.8747	-0.86 (1.56)	-0.75 (1.15)	0.1323	-0.75 (1.57)	-0.83 (1.30)	0.5938	-0.91 (1.5)	-0.99 (1.26)	0.4643

Note: (a) ANOVA (Null hypothesis: Mean BMI Z score values change was not different from IHDS-1 to IHDS-2 within the 4 groups)

(b) Statistical significance denoted by p-value<0.05, p-value<0.01, p-value<0.001, reject null hypothesis-there is a significant difference within the groups.

(c) Abbreviations: SD, Standard Deviation; MDM, Mid Day Meal; BMI, Body Mass Index; IHDS, Indian Human Development Survey; ANOVA, Analysis of Variance.

Table 5 shows the change in the asset group category of the respondents from IHDS-1 to IHDS-2 in percentages. The primary outcome was to observe whether there has been a major significant shift in the asset group category of respondents from IHDS-1 to IHDS-2. Chi-square test shows a significant association between asset group in IHDS-1 and asset group in IHDS-2. There is a small shift among respondents from the poorest asset group to the poor asset group and vice versa. Around 26% of respondents shifted from the poorest asset group category in IHDS-1 to poor asset group category in IHDS-2. Vice versa, around 31% of respondents who belonged to the poor asset group in IHDS-1, now fell into the poorest category in IHDS-2. However, the overall asset group category of respondents was consistent over time from IHDS-1 (2004–2005) to IHDS-2 (2011–2012).

**Table 5:** The percentage change in the asset group over time from IHDS-1 (2004–2005) to IHDS-2 (2011–2012).

Asset group in IHDS-1	Asset group in IHDS-2 (%)			
	Poorest	Poor	Middle	Rich
**Poorest**	62.96	26.26	9.25	1.54
**Poor**	30.94	40.09	24.57	4.41
**Middle**	8.85	23.73	47.12	20.29
**Rich**	0.54	3.57	28.09	67.80

Note: (a) The variable asset group sums 30 dichotomous items measuring household possessions and housing quality and has a Cronbach’s reliability coefficient alpha of 0.914 (unweighted). The resulting asset group variable ranges from 0 to 30, with a weighted median of 10, a weighted mean of 11.3, and a weighted standard deviation of 6.3.

(b) %: Percentage

(b) Abbreviations: IHDS, Indian Human Development Survey.

Table 6 shows the results of the logistic regression analyses of the determinants of underweight children and adolescents among poor and non-poor asset groups from the IHDS-2 (2011–2012) after controlling for change in MDM consumption status, sex, household size, education of adult members in the household, religion, place of residence and region. Regression estimates reveal that study participants belonging to the poor asset group who received MDM support at an early age (OR: 2.03; CI: 1.06 to 3.87), at a late age (OR: 3.73; CI: 1.60 to 8.69), and also who received persistent MDM support (OR: 2.43; CI: 1.21 to 4.88) had higher odds of being underweight in comparison with those who did not receive MDM support at all. The magnitude of association between underweight status and change in MDM consumption status was lowest among adolescents receiving MDM support at IHDS-1. Adolescents from the non-poor asset group who received persistent MDM support (OR: 2.09; CI: 1.34 to 3.28) were more likely to be underweight as compared to those who did not receive any MDM support. Both poor (OR: 0.47; CI: 0.33 to 0.67) and non-poor (OR: 0.55; CI: 0.41 to 0.72) girls had lower odds of being underweight in comparison to boys. Adolescents residing in the Eastern region of India were less likely to be underweight in both poor (OR: 0.47; CI: 0.29 to 0.77) and Non-poor (OR: 0.31; CI: 0.16 to 0.58) asset group categories compared with their counterparts living in the Northern region.

**Table 6:** Results of logistic regression (Odds ratio and 95% confidence interval) showing the determinants of underweight children and adolescents among poor and non-poor asset groups from IHDS-2 (2011–2012).

Independent variables	Underweight in IHDS-2			
	Poor Asset Group		Non-poor Asset Group	
	Odds ratio	95% CI	Odds ratio	95% CI
**Change in MDM consumption status**				
Group 1: No MDM support ^Ref.^				
Group 2: MDM support at IHDS-1 only	2.03*	(1.06–3.87)	1.12	(0.78–1.62)
Group 3: MDM support at IHDS-2 only	3.73**	(1.60–8.69)	1.54	(0.89–2.67)
Group 4: MDM support at both IHDS-1 and IHDS-2	2.43*	(1.21–4.88)	2.09**	(1.34–3.28)
**Sex**				
Boys ^Ref.^				
Girls	0.47***	(0.33–0.67)	0.55***	(0.41–0.72)
**Household size**				
Less than equal to 4 members ^Ref.^				
5 to 8 members	1.31	(0.85–2.00)	1.07	(0.77–1.48)
Greater than equal to 9 members	1.36	(0.69–2.68)	1.08	(0.62–1.87)
**Education of adult members in the HH**				
All are illiterate ^Ref.^				
At least one completed Primary	0.79	(0.42–1.5)	1.06	(0.48–2.37)
At least one completed Secondary	0.85	(0.57–1.26)	1.52	(0.92–2.51)
At least one completed Higher	0.67	(0.36–1.26)	1.39	(0.8–2.42)
**Religion**				
Hindu ^Ref.^				
Muslim	0.90	(0.47–1.75)	1.10	(0.71–1.71)
Christian and others	-	-	0.30	(0.09–1.00)
**Place of residence**				
Rural ^Ref.^				
Urban	1.27	(0.63–2.55)	0.81	(0.59–1.12)
**Region**				
North ^Ref.^				
Central	1.34	(0.87–2.07)	1.37	(0.87–2.15)
East	0.47**	(0.29–0.77)	0.31***	(0.16–0.58)
North-East	-	-	0.55	(0.16–1.88)
West	1.66	(0.91–3.03)	1.09	(0.75–1.57)
South	0.57	(0.24–1.36)	0.93	(0.61–1.41)

Note: (a) Model 1 and 2 are adjusted for change in MDM consumption status and sociodemographic factors such as sex, household size, Education of adult members in the household, religion, place of residence and region.

(b) Poorest and poor asset groups were combined to create Poor Asset group and middle and rich asset groups were combined to create Non-poor Asset group.

(c) Respondent was considered underweight if BMI-for-age was more than two standard deviations below (< -2SD) the WHO Child Growth Standards median.

(d) Ref. denotes reference category.

(e) Statistical significance denoted by asterisks: *, **, *** are for p-value<0.05, p-value<0.01, p-value<0.001 respectively. A significance level of 0.05 indicates a 5% risk of concluding that an association exists between the dependent and independent variables. In these results, the odds ratio of 2.03 for Group 2 from the poor asset category is statistically significant at the significance level of 0.05, therefore, Group 2 beneficiaries are 2.03 times more likely to be underweight.

(f) 95% Confidence interval is given in parenthesis.

(g) Christian and others category has very few respondents.

(h) Abbreviations: MDM, Mid-Day Meal; HH, Household; IHDS, Indian Human Development Survey.

## Discussion

In this study, we have assessed the impact of the world’s largest school feeding programme using a nationally representative data on the underweight prevalence according to the transition in MDM consumption among children and adolescents aged 6 to 9 years in IHDS-1 who then turned 13 to 16 years in IHDS-2 in India. The original and important findings indicate that the MDM programme had a positive and significant impact on lowering the underweight prevalence of the beneficiaries. However, after controlling for certain background characteristics, the odds of being underweight were significantly higher for those who received MDM support in at least one of the two survey rounds in comparison with those who did not receive MDM support at all. Children consuming MDM at younger ages (6 to 9 years) were less affected by underweight. Adolescent girls in the age group 13 to 16 years were less likely to be underweight than boys, regardless of their socioeconomic status.

Study participants from the poor asset group who received MDM support at an early age, late age, or who received persistent MDM support were more likely to be underweight as compared to those who did not receive MDM support at all. A review by Jomaa (2011) showed that previous studies have revealed mixed findings regarding how school feeding affects children’s weight, height, and BMI gains [29]. Prior research by Jacoby (2002) and Afridi (2010) focused on the nutritional intake outcomes of MDM beneficiaries, whereas Singh, Park and Dercon (2014) is the only study that focused on the outcome indicators of child nutrition i.e., the anthropometric z-scores on two measures, WFA and HFA. Also, the mean age of the study sample considered in these earlier studies was 4.7 to 8.5 years [20, 30, 31], whereas the current study covers a broader age group of children ages ranging from 6 to 9 years in IHDS-1 who then turned 13 to 16 years in IHDS-2 and attended government, government aided, EGS, and Madrassa schools. Also, the study participants when last surveyed in IHDS-2 were currently in school but may not be consuming MDM since the scheme was extended to cover children in upper primary classes (i.e., classes VI to VIII) in April 2008 [14].

The magnitude of the association between change in MDM consumption status and being underweight was lowest among adolescents receiving MDM support only in IHDS-1 (2004–05), indicating that MDMs were more effective in reducing underweight prevalence among children when consumed only at younger ages. At the time of IHDS-1 in 2004–05, children in our analytical group were in the age range of 6 to 9 years and would have received the MDM for a minimum duration of about 1 year and a maximum of 4 years. This result indicates that dietary interventions are more successful in childhood (age 6 to 9 years) than in adolescence (age 13 to 16 years) because there is potential for catch-up if conditions are made better, for instance through nutritional supplementation when children are still young [see, 32–34]. Importantly, the period between 5 and 9 years of age is a time of continued growth and development and children are affected by multiple forms of malnutrition [35]. Therefore, dietary interventions may directly alter the intake of children through the school lunch programme, since schools are key settings that provide access to a large proportion of children for prolonged periods [36, 37]. Moreover, adolescents from the non-poor asset group who received persistent MDM support were also more likely to be underweight as compared to those who did not receive any MDM support. These results, however, differ from those of other earlier research, which revealed that children from higher-income families have access to a number of healthier food options through their home meals along with the MDM provided in schools, and hence participants from the Non-poor asset group category have a better nutritional status [27, 38].

Adolescent girls aged 13 to 16 years were less likely to be underweight than boys, regardless of their socioeconomic status. These findings are in accordance with some of the previous studies worldwide and in India, which report a sex difference in the prevalence of underweight, with boys having a higher prevalence compared to girls both among children and adolescents [9, 10]. Compared to their counterparts living in the Northern area, adolescents living in India’s Eastern region were less likely to be underweight in both the poor and non-poor asset group categories. The result from the current study is consistent with a study performed using the CNNS (2016–18) data which reported that adolescents from Eastern India were at decreased odds of thinness compared to adolescents from Northern India [39].

There are some limitations to the study which should be considered. First and foremost, as these factors influence children’s underweight status, data on school-related features, including adequate water and sanitation facilities in schools, maternal characteristics, inflammation and infectious disease history, and dietary diversity could have offered more insight. Secondly, the meals provided 300 kilocalories and 8–12 gram protein per day; however, we were unable to estimate the full nutritional composition (fats, carbohydrates and micronutrients) of the MDM due to the lack of data in the survey. This may influence the effectiveness of the MDM programme in reducing the prevalence of underweight. There was a drop period (between 9 to 13 years of age) where the participants were not examined between IHDS-1 and IHDS-2, and we could not evaluate the underweight or the MDM consumption status of the children during that period. Lastly, the duration of the research participants’ MDM support would have been another crucial component to include when studying the relationship between MDM consumption status and underweight.

Undernutrition is more common in early childhood and is also likely to persist through adolescence into adulthood, and therefore, this "second opportunity" for catch-up growth during adolescence should not be missed [28]. Schools providing cooked meals are mostly government or government-aided schools where the cost of schooling is generally lower, which attracts children from the lower economic strata. There is extensive literature that states that children attending government schools and belonging to lower socioeconomic strata are more likely to be undernourished, and thus, for the vulnerable sections of the country, a scheme like this can serve its purpose [7, 28, 40]. The estimates drawn from these large datasets could help policymakers determine the extent to which operational goals are met and set priorities to facilitate target-based decision making. In continuation of this study, we would be analyzing the data from the IHDS using a Generalized Estimating Equations (GEE) model. For the MDM programme to reflect effectively on its beneficiaries, extensive use of available data for monitoring every stage of the programme through longitudinal comparison of the indicators will appropriately demonstrate the effectiveness of the intervention.

## Conclusion

This study has shown that the MDM programme administered to the beneficiaries was effective in reducing the rate of underweight (defined as BMI-for-age < - 2 SD). However, the MDM programme was not effective in reducing the underweight prevalence among beneficiaries from the poor asset group. Children consuming MDM at younger ages (6 to 9 years) were less affected by underweight. Adolescent girls in the age group 13 to 16 years were less likely to be underweight than boys, regardless of their socioeconomic status. However, continuous programme upscaling with a special focus on children from poor households will significantly benefit India’s school-aged children. Given the Indian context, this is one of the few attempts at a careful assessment of a programme using a nationally representative dataset, and these original findings, alongside with other research on the beneficial effects of school meals on school enrolment, attendance, and daily nutrient intake, offer empirical support for the advantages of the programme in India.

These findings could be taken to support a broad focus by the government, thus providing a basis for potential new policy recommendations, tackling the dual and triple burden of malnutrition, and implementing programmes in the early years to instill healthy lifelong eating habits. Moreover, this research should lead to further research focusing on individuals’ physical growth outcomes, thus giving way to sub- programmes focusing on the factors emerging from the current study. The wider applicability of these findings could help the central and state governments identify regional- and state-specific measures, thereby positively impacting growth outcomes and future adult health amongst Indian children and adolescents.

## Supporting information

S1 Table **A**. Results of logistic regression showing the determinants of underweight children and adolescents among poor asset groups from IHDS-2 (2011–2012). Note: (a) This model is adjusted for change in MDM consumption status and sociodemographic factors such as sex, household size, Education of adult members in the household, religion, place of residence and region. (b) Poorest and poor asset groups were combined to create Poor Asset group. (c) Respondent was considered underweight if BMI-for-age was more than two standard deviations below (< -2SD) the WHO Child Growth Standards median. (d) Ref. denotes reference category. (e) The z value is the ratio of the estimated coefficient to its standard error and it measures the number of standard deviations that the estimated coefficient is away from 0. (f) The P >|z| column represents the p-value for each coefficient. A significance level of 0.05 indicates a 5% risk of concluding that an association exists between the dependent and independent variables. In these results, the odds ratio of 2.03 for Group 2 is statistically significant at the significance level of 0.05, therefore, Group 2 beneficiaries are 2.03 times more likely to be underweight. (g) Christian and others category has very few respondents. (h) Abbreviations: MDM, Mid-Day Meal; HH, Household; IHDS, Indian Human Development Survey. **B**. Results of logistic regression showing the determinants of underweight children and adolescents among non-poor asset groups from IHDS-2 (2011–2012). Note: (a) This model is adjusted for change in MDM consumption status and sociodemographic factors such as sex, household size, Education of adult members in the household, religion, place of residence and region. (b) Middle and rich asset groups were combined to create Non-poor Asset group. (c) Respondent was considered underweight if BMI-for-age was more than two standard deviations below (< -2SD) the WHO Child Growth Standards median. (d) Ref. denotes reference category. (e) The z value is the ratio of the estimated coefficient to its standard error and it measures the number of standard deviations that the estimated coefficient is away from 0. (f) The P >|z| column represents the p-value for each coefficient. A significance level of 0.05 indicates a 5% risk of concluding that an association exists between the dependent and independent variables. In these results, the odds ratio of 2.09 for Group 4 is statistically significant at the significance level of 0.05, therefore, Group 4 beneficiaries are 2.09 times more likely to be underweight. (g) Abbreviations: MDM, Mid-Day Meal; HH, Household; IHDS, Indian Human Development Survey.
(DOCX)

S1 Checklist STROBE statement—Checklist of items that should be included in reports of observational studies. Note: An Explanation and Elaboration article discusses each checklist item and gives methodological background and published examples of transparent reporting. The STROBE checklist is best used in conjunction with this article (freely available on the Web sites of PLoS Medicine at http://www.plosmedicine.org/, Annals of Internal Medicine at http://www.annals.org/, and Epidemiology at http://www.epidem.com/). Information on the STROBE Initiative is available at www.strobe-statement.org.
(DOCX)
