# Supplementary material for: The effect of the Mid-Day Meal programme on the longitudinal physical growth from childhood to adolescence in India
Source: PLOS Glob Public Health. 2024 Jan 11;4(1):e0002742. doi: 10.1371/journal.pgph.0002742 (PMC10783765; doi:10.1371/journal.pgph.0002742)
Supplement: S1 Table — A. Results of logistic regression showing the determinants of underweight children and adolescents among poor asset groups from IHDS-2 (2011–2012). Note: (a) This model is adjusted for change in MDM consumption status and sociodemographic factors such as sex, household size, Education of adult members in the household, religion, place of residence and region. (b) Poorest and poor asset groups were combined to create Poor Asset group. (c) Respondent was considered underweight if BMI-for-age was more than two standard deviations below (< -2SD) the WHO Child Growth Standards median. (d) Ref. denotes reference category. (e) The z value is the ratio of the estimated coefficient to its standard error and it measures the number of standard deviations that the estimated coefficient is away from 0. (f) The P >|z| column represents the p-value for each coefficient. A significance level of 0.05 indicates a 5% risk of concluding that an association exists between the dependent and independent variables. In these results, the odds ratio of 2.03 for Group 2 is statistically significant at the significance level of 0.05, therefore, Group 2 beneficiaries are 2.03 times more likely to be underweight. (g) Christian and others category has very few respondents. (h) Abbreviations: MDM, Mid-Day Meal; HH, Household; IHDS, Indian Human Development Survey. B. Results of logistic regression showing the determinants of underweight children and adolescents among non-poor asset groups from IHDS-2 (2011–2012). Note: (a) This model is adjusted for change in MDM consumption status and sociodemographic factors such as sex, household size, Education of adult members in the household, religion, place of residence and region. (b) Middle and rich asset groups were combined to create Non-poor Asset group. (c) Respondent was considered underweight if BMI-for-age was more than two standard deviations below (< -2SD) the WHO Child Growth Standards median. (d) Ref. denotes reference category. (e) The z valu [file pgph.0002742.s001.docx]

**Supporting Information**

**S1A Table.** **Results of logistic regression showing the determinants of underweight children and adolescents among poor asset groups from IHDS-2 (2011–2012).**

| **Independent variables** | **Odds ratio** | **Robust standard error** | **z** | **P>\|z\|** | **[95% Confidence Interval]** | |
| --- | --- | --- | --- | --- | --- | --- |
| **Change in MDM consumption status** |  |  |  |  |  |  |
| Group 1: No MDM support ^Ref.^ |  |  |  |  |  |  |
| Group 2: MDM support at IHDS-1 only | 2.026858 | 0.669817 | 2.14 | 0.033 | 1.060538 | 3.87365 |
| Group 3: MDM support at IHDS-2 only | 3.727706 | 1.610565 | 3.05 | 0.002 | 1.598372 | 8.693715 |
| Group 4: MDM support at both IHDS-1 and IHDS-2 | 2.430112 | 0.8634645 | 2.5 | 0.012 | 1.211095 | 4.876118 |
| **Sex** |  |  |  |  |  |  |
| Boys ^Ref.^ |  |  |  |  |  |  |
| Girls | 0.4731244 | 0.0852677 | -4.15 | 0.000 | 0.33233 | 0.6735675 |
| **Household size** |  |  |  |  |  |  |
| Less than equal to 4 members ^Ref.^ |  |  |  |  |  |  |
| 5 to 8 members | 1.305057 | 0.2829747 | 1.23 | 0.219 | 0.8532258 | 1.996158 |
| Greater than equal to 9 members | 1.360129 | 0.4701483 | 0.89 | 0.374 | 0.6907959 | 2.677998 |
| **Education of adult members in the HH** |  |  |  |  |  |  |
| All are illiterate ^Ref.^ |  |  |  |  |  |  |
| At least one completed Primary | 0.7883578 | 0.2574285 | -0.73 | 0.466 | 0.4156943 | 1.495108 |
| At least one completed Secondary | 0.846174 | 0.1703161 | -0.83 | 0.407 | 0.570337 | 1.255416 |
| At least one completed Higher | 0.674559 | 0.2150049 | -1.24 | 0.217 | 0.3611712 | 1.259873 |
| **Religion** |  |  |  |  |  |  |
| Hindu ^Ref.^ |  |  |  |  |  |  |
| Muslim | 0.9039971 | 0.303522 | -0.3 | 0.764 | 0.4681353 | 1.745672 |
| Christian and others | - | - | - | - | - | - |
| **Place of residence** |  |  |  |  |  |  |
| Rural ^Ref.^ |  |  |  |  |  |  |
| Urban | 1.266621 | 0.452637 | 0.66 | 0.508 | 0.6287282 | 2.551704 |
| **Region** |  |  |  |  |  |  |
| North ^Ref.^ |  |  |  |  |  |  |
| Central | 1.342636 | 0.2966632 | 1.33 | 0.182 | 0.8707241 | 2.070314 |
| East | 0.4717172 | 0.1175576 | -3.02 | 0.003 | 0.2894356 | 0.7687964 |
| North-East | 1 | (empty) | - | - | - | - |
| West | 1.656537 | 0.509994 | 1.64 | 0.101 | 0.9060327 | 3.028716 |
| South | 0.5677055 | 0.2540276 | -1.27 | 0.206 | 0.236179 | 1.364599 |
| Constant | 0.1829988 | 0.0688964 | -4.51 | 0.000 | 0.087495 | 0.3827484 |
| **Wald chi-square(15)** | 49.88 | | | | | |
| **Prob > chi-square** | 0.0000 | | | | | |
| **Pseudo R2** | 0.0624 | | | | | |
| **Log pseudolikelihood** | -416.84583 | | | | | |

Note: (a) This model is adjusted for change in MDM consumption status and sociodemographic factors such as sex, household size, Education of adult members in the household, religion, place of residence and region.

(b) Poorest and poor asset groups were combined to create Poor Asset group.

(c) Respondent was considered underweight if BMI-for-age was more than two standard deviations below (< -2SD) the WHO Child Growth Standards median.

(d) Ref. denotes reference category.

(e) The z value is the ratio of the estimated coefficient to its standard error and it measures the number of standard deviations that the estimated coefficient is away from 0.

(f) The P >|z| column represents the p-value for each coefficient. A significance level of 0.05 indicates a 5% risk of concluding that an association exists between the dependent and independent variables. In these results, the odds ratio of 2.03 for Group 2 from the poor asset category is statistically significant at the significance level of 0.05, therefore, Group 2 beneficiaries are 2.03 times more likely to be underweight.

(g) Christian and others category has very few respondents.

(h) Abbreviations: MDM, Mid-Day Meal; HH, Household; IHDS, Indian Human Development Survey.

**S1B Table.** **Results of logistic regression showing the determinants of underweight children and adolescents among non-poor asset groups from IHDS-2 (2011–2012).**

| **Independent variables** | **Odds ratio** | **Robust standard error** | **z** | **P>\|z\|** | **[95% Confidence Interval]** | |
| --- | --- | --- | --- | --- | --- | --- |
| **Change in MDM consumption status** |  |  |  |  |  |  |
| Group 1: No MDM support ^Ref.^ |  |  |  |  |  |  |
| Group 2: MDM support at IHDS-1 only | 1.124267 | 0.2111453 | 0.62 | 0.533 | 0.7780502 | 1.624542 |
| Group 3: MDM support at IHDS-2 only | 1.538606 | 0.4312575 | 1.54 | 0.124 | 0.8882665 | 2.665088 |
| Group 4: MDM support at both IHDS-1 and IHDS-2 | 2.094736 | 0.4776337 | 3.24 | 0.001 | 1.339805 | 3.275043 |
| **Sex** |  |  |  |  |  |  |
| Boys ^Ref.^ |  |  |  |  |  |  |
| Girls | 0.5452642 | 0.0783356 | -4.22 | 0.000 | 0.4114517 | 0.7225953 |
| **Household size** |  |  |  |  |  |  |
| Less than equal to 4 members ^Ref.^ |  |  |  |  |  |  |
| 5 to 8 members | 1.065648 | 0.1781065 | 0.38 | 0.704 | 0.7679776 | 1.478696 |
| Greater than equal to 9 members | 1.078544 | 0.3031634 | 0.27 | 0.788 | 0.6216951 | 1.871107 |
| **Education of adult members in the HH** |  |  |  |  |  |  |
| All are illiterate ^Ref.^ |  |  |  |  |  |  |
| At least one completed Primary | 1.06154 | 0.4340459 | 0.15 | 0.884 | 0.4763148 | 2.365802 |
| At least one completed Secondary | 1.520904 | 0.3879216 | 1.64 | 0.100 | 0.9225592 | 2.507316 |
| At least one completed Higher | 1.393329 | 0.3916863 | 1.18 | 0.238 | 0.8030965 | 2.41735 |
| **Religion** |  |  |  |  |  |  |
| Hindu ^Ref.^ |  |  |  |  |  |  |
| Muslim | 1.102217 | 0.2482904 | 0.43 | 0.666 | 0.7087967 | 1.714007 |
| Christian and others | 0.2975527 | 0.1836569 | -1.96 | 0.050 | 0.0887526 | 0.9975772 |
| **Place of residence** |  |  |  |  |  |  |
| Rural ^Ref.^ |  |  |  |  |  |  |
| Urban | 0.8138248 | 0.1310659 | -1.28 | 0.201 | 0.5935339 | 1.115877 |
| **Region** |  |  |  |  |  |  |
| North ^Ref.^ |  |  |  |  |  |  |
| Central | 1.36919 | 0.315935 | 1.36 | 0.173 | 0.871069 | 2.152163 |
| East | 0.3054043 | 0.0998685 | -3.63 | 0.000 | 0.1608898 | 0.5797245 |
| North-East | 0.5535855 | 0.3448744 | -0.95 | 0.343 | 0.1632678 | 1.87702 |
| West | 1.086481 | 0.2047486 | 0.44 | 0.660 | 0.7509524 | 1.571926 |
| South | 0.9285921 | 0.1979103 | -0.35 | 0.728 | 0.6115175 | 1.410071 |
| Constant | 0.1759579 | 0.054665 | -5.59 | 0.000 | 0.0957117 | 0.3234838 |
| **Wald chi-square (15)** | 61.98 | | | | | |
| **Prob > chi-square** | 0.0000 | | | | | |
| **Pseudo R2** | 0.0516 | | | | | |
| **Log pseudolikelihood** | -650.30593 | | | | | |

Note: (a) This model is adjusted for change in MDM consumption status and sociodemographic factors such as sex, household size, Education of adult members in the household, religion, place of residence and region.

(b) Middle and rich asset groups were combined to create Non-poor Asset group.

(c) Respondent was considered underweight if BMI-for-age was more than two standard deviations below (< -2SD) the WHO Child Growth Standards median.

(d) Ref. denotes reference category.

(e) The z value is the ratio of the estimated coefficient to its standard error and it measures the number of standard deviations that the estimated coefficient is away from 0.

(f) The P >|z| column represents the p-value for each coefficient. A significance level of 0.01 indicates a 1% risk of concluding that an association exists between the dependent and independent variables. In these results, the odds ratio of 2.09 for Group 4 is statistically significant at the significance level of 0.01, therefore, Group 4 beneficiaries are 2.09 times more likely to be underweight.

(g) Abbreviations: MDM, Mid-Day Meal; HH, Household; IHDS, Indian Human Development Survey.
