# Supplementary material for: The effect of the Mid-Day Meal programme on the longitudinal physical growth from childhood to adolescence in India
Source: PLOS Glob Public Health. 2024 Jan 11;4(1):e0002742. doi: 10.1371/journal.pgph.0002742 (PMC10783765; doi:10.1371/journal.pgph.0002742)
Supplement: S1 Checklist — Note: An Explanation and Elaboration article discusses each checklist item and gives methodological background and published examples of transparent reporting. The STROBE checklist is best used in conjunction with this article (freely available on the Web sites of PLoS Medicine at http://www.plosmedicine.org/, Annals of Internal Medicine at http://www.annals.org/, and Epidemiology at http://www.epidem.com/). Information on the STROBE Initiative is available at www.strobe-statement.org. (DOCX) [file pgph.0002742.s002.docx]

**Supporting Information**

**S1 Checklist. STROBE Statement—checklist of items that should be included in reports of observational studies.**

|  | Item No. | Recommendation | Page  No. | Relevant text from manuscript |
| --- | --- | --- | --- | --- |
| **Title and abstract** | 1 | (*a*) Indicate the study’s design with a commonly used term in the title or the abstract | 1-2 | This longitudinal study utilised data from the Indian Human Development Survey (IHDS) Rounds 1 (2004-05) and 2 (2011-12) to study the effect of the Mid-Day Meal programme on changes in the underweight prevalence among children and adolescents in India. |
|  |  | (*b*) Provide in the abstract an informative and balanced summary of what was done and what was found | 2 | Lines (28-47); Abstract |
| Introduction | | | |  |
| Background/rationale | 2 | Explain the scientific background and rationale for the investigation being reported | 3-5 | Lines (52-110); Introduction |
| Objectives | 3 | State specific objectives, including any prespecified hypotheses | 5 | Lines (104-110); Employing a longitudinal data set from Rounds 1 and 2 of the Indian Human Development Survey (IHDS-1 and 2), the present study aimed to examine the changes in the prevalence of underweight (defined as BMI-for-age < -2 SD of the WHO Child Growth Standards median) in schoolchildren aged 6 to 9 years in IHDS-1 and 13 to 16 years in IHDS-2 transition from MDM beneficiaries to non-beneficiaries and vice versa. The secondary aim was to examine the prevalence of underweight status according to different socio-demographic characteristics and determine their predictors. |
| Methods | | | |  |
| Study design | 4 | Present key elements of study design early in the paper | 6 | Lines (139-140); We utilized panel data from Rounds 1 (2004-05) and 2 (2011-12) of the IHDS, and our final sample size was 3,199 (1,638 girls and 1,561 boys). |
| Setting | 5 | Describe the setting, locations, and relevant dates, including periods of recruitment, exposure, follow-up, and data collection | 5-6 | Lines (120-136); Materials and methods; Data source |
| Participants | 6 | (*a*) *Cohort study*—Give the eligibility criteria, and the sources and methods of selection of participants. Describe methods of follow-up  *Case-control study*—Give the eligibility criteria, and the sources and methods of case ascertainment and control selection. Give the rationale for the choice of cases and controls  *Cross-sectional study*—Give the eligibility criteria, and the sources and methods of selection of participants | 6 | Lines (139-143); We utilized panel data from Rounds 1 (2004-05) and 2 (2011-12) of the IHDS, and our final sample size was 3,199 (1,638 girls and 1,561 boys). For the analytical purposes of our study, data was restricted to the individual-level information of children aged 6 to 9 years in IHDS-1 who then turned 13 to 16 years in IHDS-2 and were currently attending government, government-aided, EGS, and Madrassa schools. |
|  |  | (*b*) *Cohort study*—For matched studies, give matching criteria and number of exposed and unexposed  *Case-control study*—For matched studies, give matching criteria and the number of controls per case |  |  |
| Variables | 7 | Clearly define all outcomes, exposures, predictors, potential confounders, and effect modifiers. Give diagnostic criteria, if applicable | 7 | Lines (161-182); Variable description |
| Data sources/ measurement | 8* | For each variable of interest, give sources of data and details of methods of assessment (measurement). Describe comparability of assessment methods if there is more than one group | 5-7 | Lines (121-182); Data source, Study design, Variable description |
| Bias | 9 | Describe any efforts to address potential sources of bias |  |  |
| Study size | 10 | Explain how the study size was arrived at | 6 | Lines (138-152); Study design.  Lines (155-158); Details of the final sample of re-interviewed respondents and the sample selection process are presented in Fig 1. |

Continued on next page

| Quantitative variables | 11 | Explain how quantitative variables were handled in the analyses. If applicable, describe which groupings were chosen and why | 7-8 | Lines (161-182); Variable description  Lines (189-193); Chi-square tests were used to determine whether independent variables, sex, household size, asset group, household adult’s education, place of residence, religion and region had significant associations with the prevalence of underweight status at p < 0.05. The asset group has not changed over time from IHDS-1 (2004–2005) to IHDS-2 (2011–2012) which is why we have considered only the sample from IHDS-2 (2011–2012) in our multivariate analysis. |
| --- | --- | --- | --- | --- |
| Statistical methods | 12 | (*a*) Describe all statistical methods, including those used to control for confounding | 8 | Lines (184-208); Statistical analyses |
|  |  | (*b*) Describe any methods used to examine subgroups and interactions | 8 | Lines (203-205); Analysis of variance (ANOVA) was used to determine significant change in BMI Z scores from IHDS-1 to IHDS-2 within the four groups and whether that change was different between the groups. |
|  |  | (*c*) Explain how missing data were addressed |  | The study sample had very few missing values; therefore, we excluded missing cases from the study sample. |
|  |  | (*d*) *Cohort study*—If applicable, explain how loss to follow-up was addressed  *Case-control study*—If applicable, explain how matching of cases and controls was addressed  *Cross-sectional study*—If applicable, describe analytical methods taking account of sampling strategy | 6 | Lines (148-150); The percentage of loss to follow-up for all the observations was 17%, and for our study sample it was 25%, which was very low; therefore, we excluded individuals lost to recontact for IHDS-2 from the study sample. |
|  |  | (*e*) Describe any sensitivity analyses | N/A |  |
| Results | | | | |
| Participants | 13* | (a) Report numbers of individuals at each stage of study—eg numbers potentially eligible, examined for eligibility, confirmed eligible, included in the study, completing follow-up, and analysed |  | Included in Fig 1 and submitted along with the online application |
|  |  | (b) Give reasons for non-participation at each stage | N/A |  |
|  |  | (c) Consider use of a flow diagram |  | Included in Fig 1 and submitted along with the online application |
| Descriptive data | 14* | (a) Give characteristics of study participants (eg demographic, clinical, social) and information on exposures and potential confounders | 9-10 | Lines (222); Table 1. Socio-demographic characteristics and growth outcomes of the study population, IHDS-1 (2004–2005) and IHDS-2 (2011–2012) (N=3,199) |
|  |  | (b) Indicate number of participants with missing data for each variable of interest | 9-10 | Lines (222); Table 1. Socio-demographic characteristics and growth outcomes of the study population, IHDS-1 (2004–2005) and IHDS-2 (2011–2012) (N=3,199)  Education of adult members in the household variable had missing data for 5 study participants in IHDS-1 (N=3,194 in IHDS-1) |
|  |  | (c) *Cohort study*—Summarise follow-up time (eg, average and total amount) |  |  |
| Outcome data | 15* | *Cohort study*—Report numbers of outcome events or summary measures over time | 11-13 | Lines (240); Table 2. Underweight prevalence among school-aged children by socioeconomic characteristics and change in MDM consumption status from IHDS-1 (2004–2005) to IHDS-2 (2011–2012) (N=3,199) |
|  |  | *Case-control study—*Report numbers in each exposure category, or summary measures of exposure | N/A |  |
|  |  | *Cross-sectional study—*Report numbers of outcome events or summary measures | N/A |  |
| Main results | 16 | (*a*) Give unadjusted estimates and, if applicable, confounder-adjusted estimates and their precision (eg, 95% confidence interval). Make clear which confounders were adjusted for and why they were included | 13-18 | Lines (255); Table 3. Analysis of variance for BMI Z scores in IHDS-2 and MDM consumption status from IHDS-1 to IHDS-2  Lines (264); Table 4. Analysis of variance for BMI Z scores by sex and change in MDM consumption status from IHDS-1 to IHDS-2  Lines (276); Table 5. The percentage change in the asset group over time from IHDS-1 (2004–2005) to IHDS-2 (2011–2012).  Lines (298); Table 6. Results of logistic regression (Odds ratio and 95% confidence interval) showing the determinants of underweight children and adolescents among poor and non-poor groups from IHDS-2 (2011–2012). |
|  |  | (*b*) Report category boundaries when continuous variables were categorized | 7 | Lines (166-169); Data on the height and weight of the respondents were recorded in IHDS-1, and followed up in IHDS-2. BMI-for-age Z-scores were calculated, and the results were classified as underweight if their BMI-for-age was more than two standard deviations below (< -2 SD) the WHO Child Growth Standards median. |
|  |  | (*c*) If relevant, consider translating estimates of relative risk into absolute risk for a meaningful time period | NA |  |

Continued on next page

| Other analyses | 17 | Report other analyses done—eg analyses of subgroups and interactions, and sensitivity analyses | 11-18 | Lines (240); Table 2. Underweight prevalence among school-aged children by socioeconomic characteristics and change in MDM consumption status from IHDS-1 (2004–2005) to IHDS-2 (2011–2012) (N=3,199)  Lines (255); Table 3. Analysis of variance for BMI Z scores in IHDS-2 and MDM consumption status from IHDS-1 to IHDS-2  Lines (264); Table 4. Analysis of variance for BMI Z scores by sex and change in MDM consumption status from IHDS-1 to IHDS-2  Lines (276); Table 5. The percentage change in the asset group over time from IHDS-1 (2004–2005) to IHDS-2 (2011–2012).  Lines (298); Table 6. Results of logistic regression (Odds ratio and 95% confidence interval) showing the determinants of underweight children and adolescents among poor and non-poor groups from IHDS-2 (2011–2012). |
| --- | --- | --- | --- | --- |
| Discussion | | | | |
| Key results | 18 | Summarise key results with reference to study objectives | 18-21 | Lines (318-390); Discussion  Lines (393-411); Conclusion |
| Limitations | 19 | Discuss limitations of the study, taking into account sources of potential bias or imprecision. Discuss both direction and magnitude of any potential bias | 18-21 | Lines (318-390); Discussion |
| Interpretation | 20 | Give a cautious overall interpretation of results considering objectives, limitations, multiplicity of analyses, results from similar studies, and other relevant evidence | 18-21 | Lines (318-390); Discussion |
| Generalisability | 21 | Discuss the generalisability (external validity) of the study results | 18-21 | Lines (319-322); In this study, we have assessed the impact of the world’s largest school feeding programme using a nationally representative data on the underweight prevalence according to the transition in MDM consumption among children and adolescents aged 6 to 11 years in IHDS-1 who then turned 13 to 18 years in IHDS-2 in India.  Lines (378-390); Undernutrition is more common in early childhood and is also likely to persist through adolescence into adulthood, and therefore, this "second opportunity" for catch-up growth during adolescence should not be missed [28]. Schools providing cooked meals are mostly government or government-aided schools where the cost of schooling is generally lower, which attracts children from the lower economic strata. There is extensive literature that states that children attending government schools and belonging to lower socioeconomic strata are more likely to be undernourished, and thus, for the vulnerable sections of the country, a scheme like this can serve its purpose [28, 40-41]. The estimates drawn from these large datasets could help policymakers determine the extent to which operational goals are met and set priorities to facilitate target-based decision making. For the MDM programme to reflect effectively on its beneficiaries, extensive use of available data for monitoring every stage of the programme through longitudinal comparison of the indicators will appropriately demonstrate the effectiveness of the intervention.  Lines (400-404); Given the Indian context, this is one of the few attempts at a careful assessment of a programme using a nationally representative dataset, and these original findings, alongside with other research on the beneficial effects of school meals on school enrolment, attendance, and daily nutrient intake, offer empirical support for the advantages of the programme in India. |
| Other information | |  | | |
| Funding | 22 | Give the source of funding and the role of the funders for the present study and, if applicable, for the original study on which the present article is based |  | Submitted along with the online application form |

*Give information separately for cases and controls in case-control studies and, if applicable, for exposed and unexposed groups in cohort and cross-sectional studies.

**Note:** An Explanation and Elaboration article discusses each checklist item and gives methodological background and published examples of transparent reporting. The STROBE checklist is best used in conjunction with this article (freely available on the Web sites of PLoS Medicine at http://www.plosmedicine.org/, Annals of Internal Medicine at http://www.annals.org/, and Epidemiology at http://www.epidem.com/). Information on the STROBE Initiative is available at www.strobe-statement.org.
